# Aripiprazole Attenuates Cognitive Impairments Induced by Lipopolysaccharide in Rats through the Regulation of Neuronal Inflammation, Oxidative Stress, and Apoptosis

**DOI:** 10.3390/medicina60010046

**Published:** 2023-12-26

**Authors:** Vasudevan Mani, Bander Shehail Alshammeri

**Affiliations:** 1 Department of Pharmacology and Toxicology, College of Pharmacy, Qassim University, Buraydah 51452, Saudi Arabia; 2 Pharmacy Department, Maternity and Children Hospital, Qassim Cluster, Ministry of Health, Buraydah 52384, Saudi Arabia; bandershe3@gmail.com

**Keywords:** aripiprazole, lipopolysaccharide, recognition memory, inflammation, oxidative stress, apoptosis

## Abstract

*Background and Objectives*: Aripiprazole (APZ), an atypical antipsychotic, is mainly prescribed for conditions such as schizophrenia and bipolar disorder, while ongoing research indicates promising neuroprotective qualities. APZ’s mechanism of action, involving the regulation of neurotransmitter levels, appears to contribute to its potential to shield neural tissues from specific forms of harm and degeneration. *Materials and Methods*: To investigate its neuroprotective mechanisms, groups of rats were orally administered APZ at 1 or 2 mg/kg once daily for a 30-day period. In addition, neuronal toxicity was induced through intraperitoneal injection of four doses of lipopolysaccharide (LPS) at a concentration of 1 mg/kg. To evaluate cognitive function, particularly, short-term recognition memory, the procedure implemented the novel object recognition (NOR) task. Subsequently, brain tissues were gathered to examine markers linked with neuroinflammation, oxidative stress, and apoptosis. *Results*: The administration of LPS led to a decline in memory performance during the NOR tasks. Simultaneously, this LPS treatment raised inflammatory markers like cyclooxygenase (COX)-2, tumor necrosis factor (TNF)-α, and nuclear factor kappa B (NF-κB), increased oxidative markers such as malondialdehyde (MDA), and triggered apoptosis markers like Caspase-3 and Bcl2 associated X protein (Bax) within the brain. Furthermore, it decreased levels of antioxidants like reduced glutathione (GSH) and catalase, as well as the anti-apoptotic marker B-cell lymphoma (Bcl)-2 in brain tissue. The use of APZ resulted in enhanced recognition memory performance, as indicated by improved exploration and discrimination abilities of the objects in the NOR task. Moreover, APZ lowered the markers associated with neuronal vulnerability, such as COX-2, NF-κB, MDA, Caspase-3, and Bax. Additionally, it increased the levels of protective markers, including GSH, catalase, and Bcl-2 in LPS-challenged brains. *Conclusions*: In summary, the findings suggest that APZ exhibits protective properties against neuronal inflammation, oxidative stress, and apoptosis markers in the context of inflammatory-related neurodegeneration. Additional in-depth investigations are needed to further explore potential applications.

## 1. Introduction

Neuroinflammation and neurodegeneration are intricately interconnected processes in the central nervous system. In neurons, inflammation is triggered as a part of the brain’s immune response to injury or infection, entailing the activation of microglia and the release of inflammatory molecules. Initially serving a protective function, chronic neuroinflammation can transition into a harmful state, actively contributing to the progression of neurodegeneration, which is a fundamental precursor to the development of dementia [1]. Dementia poses a significant global public health issue, impacting millions of people across the world. Moreover, it has a profound impact on a person’s daily functioning and results in cognitive deficits, encompassing issues with memory, speech, language, and communication [2]. In addition to neuronal inflammation, various factors contribute significantly to cognitive deficits in dementia. One key factor is oxidative stress, which plays a central role, and there is a significant relationship between apoptosis and neuroinflammation. These processes are intricately linked, with oxidative stress having the potential to trigger apoptosis, commonly known as cell death. Additionally, neuroinflammation can exacerbate this process by generating pro-inflammatory cytokines and ROS [3]. Given the multifaceted nature of dementia’s root causes, a comprehensive strategy for prevention and treatment is likely necessary. This strategy may involve addressing neuroinflammation, oxidative stress, apoptosis, and other relevant factors.

Epidemiological and pathophysiological research shows that in conjunction with cognitive decline, the vast majority of individuals with dementia, almost 90%, exhibit a range of behavioral symptoms, such as psychosis, agitation, aggression, and depression. Further, psychotic disorders and dementia are distinct clinical conditions that present similarities in their clinical manifestations, notably involving cognitive decline and behavioral changes [4]. Psychotic symptoms, like delusions and hallucinations, can manifest in individuals with dementia. This is commonly denoted as “psychosis in dementia”. These symptoms may arise as a consequence of the underlying structural brain changes associated with dementia, as observed in neurodegenerative conditions like Alzheimer’s disease (AD). In this setting, dementia can contribute to the emergence of psychotic characteristics [5]. In a clinical context, both typical and atypical antipsychotic agents are frequently prescribed off-label to manage psychotic symptoms in individuals with dementia, regardless of the specific subtype of dementia. It is important to recognize that antipsychotic medications may provide limited short-term relief for psychosis related to dementia [6].

Aripiprazole (APZ), an atypical antipsychotic medication, is used to treat a range of mental health disorders, including schizophrenia, autism spectrum disorder, bipolar disorder, and major depressive disorder [7]. It is well-regarded due to its effectiveness and a relatively low risk of side effects, particularly, extrapyramidal symptoms, when compared to other antipsychotic drugs [8]. Concerning its antipsychotic mechanism, APZ is known for its notable binding affinity to several receptors, including serotonin receptors like 5-HT_1A_, 5-HT_2A_, and 5-HT_2B_, as well as dopamine receptors such as D_2_ and D_3_ [7]. This unique receptor profile plays a significant role in its therapeutic effects and sets it apart from other antipsychotic medications. As previously discussed, cognitive deficits, including memory impairment, are recognized as fundamental characteristics of schizophrenia. Several observational studies have suggested that the antipsychotic medication APZ may have a positive impact on the cognitive impairment experienced by individuals with schizophrenia. These studies indicated that APZ has the potential to enhance cognitive functioning in this patient population. [9,10,11]. Further, recent research provides evidence for APZ’s capacity to improve memory functions in a range of animal models exhibiting induced memory deficits [12,13,14,15].

Neuroinflammation has been linked to the development of numerous neurological conditions. APZ offers anti-inflammatory benefits, both alone and when used in combination with other antipsychotic medications, leading to a reduction in various inflammatory parameters, including vulnerable inflammatory cytokines like TNF-α and interleukins (ILs), in individuals with schizophrenia [16,17]. Moreover, the stimulation of APZ has been noted to down-regulate the mRNA levels of IL-1β, TNF-α, and IL-6 in human immune cells [18]. Furthermore, in a rat model exposed to stress, treatment with APZ has been linked to a decrease in peripheral IL-6 levels and a reduction in hippocampal cell death [19]. Moreover, it has been proposed that oxidative stress and apoptosis may be involved in the onset of certain neurological conditions, such as dementia and schizophrenia [20]. Based on multiple recent investigations, in both in vitro and in vivo settings, APZ has exhibited antioxidant capabilities [13,21,22]. Fortunately, APZ treatment successfully reinstated diminished levels of glutathione (GSH) and catalase (CAT) induced by valproic acid in rats, while also reversing the elevated malondialdehyde (MDA) levels [21]. At the same time, APZ mitigated the reduction in brain GSH levels triggered by streptozotocin (STZ) and reduced levels of thiobarbituric acid reactive substances (TBARS) in mice [13]. Furthermore, it is essential to recognize that neuronal apoptosis is strongly correlated with neuroinflammation and oxidative stress. In this context, the pretreatment of APZ has been shown to effectively inhibit the apoptosis of oligodendrocytes in a co-culture model that includes IFN-γ-activated microglia [23]. In collective consideration, our hypothesis posited that APZ could alleviate neuronal damage induced by lipopolysaccharide (LPS)-related brain inflammation, cellular apoptosis, and increased susceptibility to oxidative stress. In order to put this hypothesis to the test, our current research project was carefully designed to explore the potential benefits of APZ in mitigating LPS-induced neurotoxicity. This encompassed an extensive examination of cognitive deficits, susceptibility to oxidative stress, neuronal inflammation, and the apoptosis process in a rat model.

## 2. Materials and Methods

### 2.1. Animals

The research protocols in the present study, which involve animal use, were ethically approved by the Deanship of Scientific Research at Qassim University (Project Number: 23-20-14; 12 January 2023). In this investigation, 24 adult male rats (Sprague Dawley; 150–200 g; 3 months) were used. To ensure randomization and experimental control, the collected rats were evenly distributed among four groups (*n* = 6). The rats were kept in polypropylene cages, with three rats accommodated in each cage. The facility maintained a temperature of 22 °C (±1) and humidity levels between 45% and 55%.

### 2.2. Vehicle

**Figure 1:**
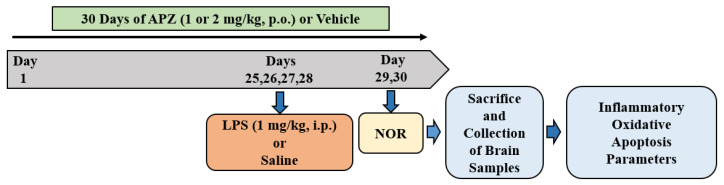
Timeline of the drug treatment and experiments. Animals received either a vehicle or APZ (1 or 2 mg/kg, p.o.) for 30 days. All animals, except those in the control group, were subjected to neurotoxicity induction using LPS (1 mg/kg, i.p; 4 doses: days 25–28). The novel object recognition (NOR) assessments were performed on day 29 (habituation) and day 30 (training and retention). Subsequently, on day 30, the brain tissues were collected for ELISA tests.

This study utilized APZ from Tabuk Pharmaceutical in Tabuk, Saudi Arabia, and formulated it as an oral suspension in a 0.5% *w*/*v* carboxymethyl cellulose solution (vehicle) for a 30-day treatment regimen. Simultaneously, LPS, procured from Sigma-Aldrich (St. Louis, MO, USA), was diluted with normal saline (NS) and delivered via intraperitoneal injections (i.p.) on days 25 through 28, as illustrated in Figure 1.

### 2.3. Experimental Design

The drug administration and maze procedures were conducted over a period of thirty days, following the experimental schedule (Figure 1). The rats were segregated into four groups as follows: control (vehicle + normal saline), LPS-induced (vehicle+ LPS, 1 mg/kg, i.p.), APZ1 + LPS (aripiprazole 1 mg/kg+ LPS), and APZ2 + LPS (aripiprazole 2 mg/kg+ LPS). The choice of APZ doses at 1 and 2 mg/kg [14,15] and the schedule for inducing LPS-related neurotoxicity were determined based on insights from early publications [24]. To evaluate cognitive performance, all animals were allowed to participate in the NOR test on days 29 and 30. The NOR test encompassed three stages: habituation, training, and the actual testing phase. Subsequent to the NOR test, all animals were humanely euthanized on the same day, and their brain tissues were harvested for ELISA analysis (Figure 1).

### 2.4. Novel Object Recognition (NOR)

The NOR test is a commonly employed behavioral assessment used with experimental animals to evaluate recognition memory. The NOR apparatus is square-shaped with all sides measuring 80 cm and with a height of 40 cm. The test was structured into three phases, which were conducted on days 29 and 30. During the habituation phase on day 29, each rat was placed in an empty box devoid of any objects for five minutes. A day later, during the training phase on day 30, rats were individually placed into a cage containing two identical items known as familiar objects (FO1 and FO2) for a five-minute interval. This allowed the rats to become acquainted with these objects. In the second part of the experiment (retention phase), which took place three hours later on the same day, one of the FOs was substituted with a novel object (NO). The rats’ behavior was observed for five minutes, during which the time spent exploring both the FO and the NO was recorded. The exploration time was determined as “the period during which an animal directs its nose toward an object located within a distance of 2 cm or less”. To assess the rat’s ability to discriminate between FOs and NOs, the discrimination index (DI% = (ENO − EFO/ENO + EFO) × 100; ENO—Exploration time of an NO; EFO—Exploration time of an FO) was computed [24,25].

### 2.5. Brain Samples Collection and Preparation of Tissue Homogenate

Upon concluding the experiment on day 30, all the rats were humanely euthanized through cervical dislocation after minimal ether anesthesia. The procedure for brain collection involved homogenizing the brains in ice-cold phosphate-buffered saline at a pH of 7.4 and a temperature of 4 degrees Celsius. The resulting homogenate was then centrifuged at 4000 rpm for ten minutes and subsequently stored at −80 degrees Celsius for later examination. The determination of the total protein content in each sample was performed utilizing the BCA assay technique.

### 2.6. Analysis of Neuroinflammation Cytokines in Brain Homogenate

Inflammatory cytokines, such as cyclooxygenase-2 (COX-2; MBS160196), tumor necrosis factor-α (TNF-α; MBS824824), and nuclear factor kappa B (NF-κB; MBS764450), were analyzed using ELISA kits obtained from MyBioSources Inc. (San Diego, CA, USA). These cytokines are associated with inflammatory processes, and each one was assessed through a specific assay protocol. The microplate was then subjected to absorbance measurement at 450 nm using a BioTek (Santa Clara, CA, USA) instrument.

### 2.7. Measurement of Oxidative Parameters in Brain Homogenate

Sandwich ELISA assay kits were utilized to quantity the levels of malondialdehyde (MDA; MBS268427), reduced glutathione (GSH; MBS265966), and catalase (MBS2704433), following the manufacturer’s guidelines as provided by MyBioSources Inc. (San Diego, CA, USA).

### 2.8. Determination of Apoptotic Proteins in Brain Homogenate

Specialized sandwich ELISA kits, referring to B-cell lymphoma-2 (Bcl-2; MBS452319), Bcl2 associated X protein (Bax; MBS2703209), and Caspase-3 (MBS261814), were employed to quantify protein levels in brain homogenate. These kits were procured from MyBioSources Inc. (San Diego, CA, USA).

### 2.9. Statistical Analysis

The data were represented as the mean value along with the standard error (SEM). Statistical analysis of the collected data was conducted using GraphPad Prism (9.5.0 version, GraphPad Software Inc., San Diego, CA, USA). To determine the levels of statistical significance between the groups, each dataset underwent a one-way ANOVA and a Tukey–Kramer post hoc test. In the NOR test, an unpaired ‘*t*’ test was utilized to compare the matching groups of the EFO and the ENO. A significance level lower than 0.05 was considered.

## 3. Results

### 3.1. Effect of APZ on Cognitive Performance in NOR Parameters

**Figure 2:**
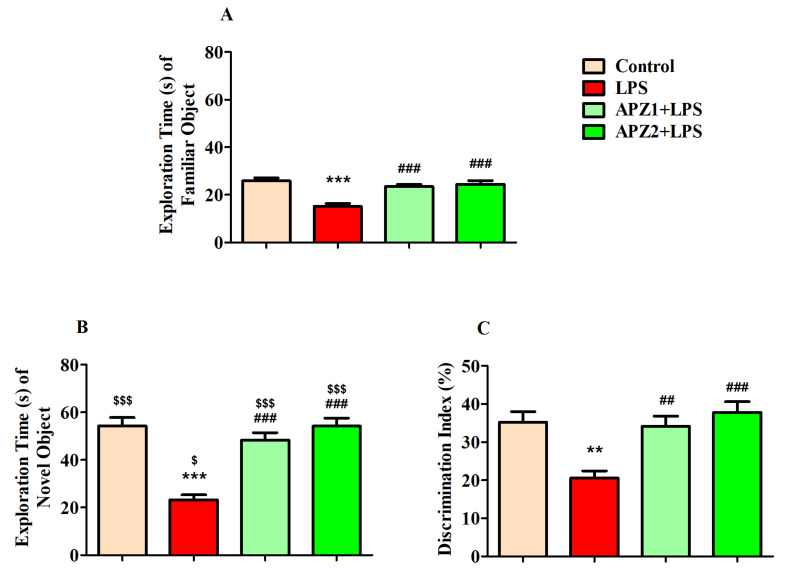
Effect of aripiprazole (APZ) on (**A**) EFO, (**B**) ENO, and (**C**) DI% of LPS-induced rats (mean ± SEM, *n* = 6) during the retention phase of a NOR test. ^$^ *p* < 0.05 and ^$$$^ *p* < 0.001 vs. corresponding groups between EFO vs. ENO; ** *p* < 0.01 and *** *p* < 0.001 vs. control group; ^##^ *p* < 0.01 and ^###^ *p* < 0.001 vs. LPS-induced group.

The research investigated the influence of APZ at two distinct dosage levels (1 or 2 mg/kg, p.o.) in conjunction with LPS on both the ENO and the EFO, along with the DI% between these objects during the test session as illustrated in Figure 2A–C, respectively.

During the initial assessment of the exploration time in the training phase for both FOs (EFO1 and EFO2), there were no significant variations detected between the objects in the control (30.67 ± 2.789 S vs. 34.33 ± 1.994 S), LPS (21.50 ± 1.996 S vs. 19.67 ± 3.138 S), APZ1 + LPS (28.17 ± 2.574 S vs. 26.83 ± 3.619 S), and APZ2 + LPS (33.50 ± 4.161 S vs. 31.17 ± 3.563 S) groups.

Considering the retention phase, in regard to the EFO, the statistical analysis revealed a substantial distinction (F(3,20) = 17.46, *p* < 0.001) across all experimental groups. As predicted, the induction of LPS resulted (15.17 ± 1.167 S) in a noteworthy decline in the EFO (*p* < 0.001) in contrast to the control that received the vehicle treatment (25.83 ± 1.327 S). This decrease was indicative of the cognitive impairment induced by LPS in the rats (LPS-induced). It is worth emphasizing that treatment with APZ at both 1 and 2 mg/kg doses (APZ1 + LPS and APZ2 + LPS, respectively), administered orally, led to a substantial and statistically significant increase (*p* < 0.001) in the EFO (23.50 ± 0.9220 S and 24.33 ± 1.606 S, respectively), as depicted in Figure 2A. This suggests that the administration of APZ effectively alleviated the cognitive impairment induced by LPS, thereby enhancing the rats’ exploration of a familiar object.

Concerning the ENO, the combined influence of APZ and LPS yielded significant distinctions (F(3,20) = 23.24, *p* < 0.001) among the various treatment groups, as depicted in Figure 2B. Rats exposed to LPS exhibited a marked reduction in the ENO (23.17 ± 2.182 S; *p* < 0.001) in contrast to the control rats (54.33 ± 3.412 S). Notably, the gavage with APZ, both at 1 and 2 mg/kg via oral administration, effectively counteracted the decrease in the ENO (48.33 ± 3.106 S and 54.17 ± 3.429 S, respectively), restoring it to levels closely resembling those in the control group, and significantly improving the ENO (*p* < 0.001) for the LPS-induced group.

An unpaired ‘*t*’ test was employed to identify the difference in the exploration time of the FO and NO between matching groups (Figure 2A,B). Except for the LPS-induced group, the groups exhibited an increase (*p* < 0.001) in the ENO parallel to the EFO. The LPS-induced groups presented a significant variation, with *p* < 0.05.

This study also examined how APZ and LPS influenced the rats’ capability to differentiate between the FO and the NO by assessing the DI% as shown in Figure 2C. Significantly, there were variations observed among the treatment groups (F(3,20) = 9.290, *p* < 0.001) with respect to DI (%). Additionally, the animals displayed a substantial decrease (20.55 ± 1.878%; *p* < 0.01) in DI (%) after being exposed to LPS, in comparison to the control group (35.22 ± 2.760%). Positively, the decline in DI (%) was effectively mitigated by the administration of APZ at both 1 mg/kg (34.15 ± 2.646%; *p* < 0.01) and 2 mg/kg (37.82 ± 2.772%; *p* < 0.001.

### 3.2. Evaluating the Influence of APZ and LPS on Neuroinflammatory Parameters

**Figure 3:**
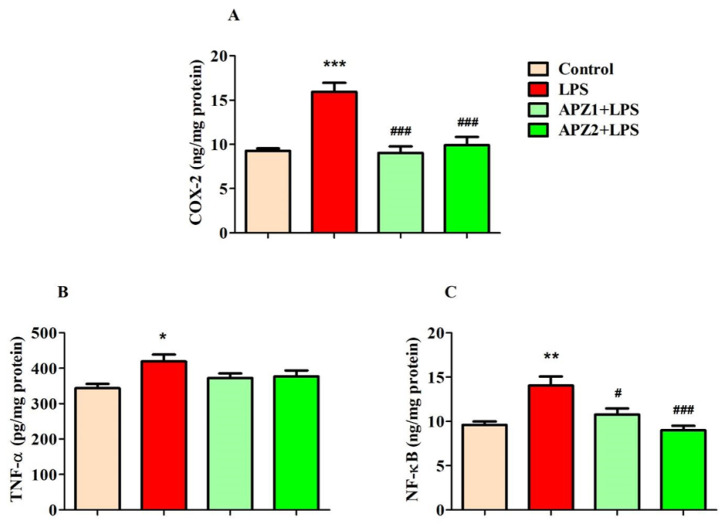
Effect of aripiprazole (APZ) on neuroinflammatory markers (**A**) COX-2, (**B**) TNF-α, and (**C**) NF-κB in LPS-induced rats (mean ± SEM, *n* = 6). * *p* < 0.05, ** *p* < 0.01, and *** *p* < 0.001 vs. control group; ^#^ *p* < 0.05, and ^###^ *p* < 0.001 vs. LPS-induced group.

Figure 3A–C depict the effects of APZ and LPS on specific inflammatory parameters in the brain.

In Figure 3A, the influence of both treatments on COX-2 levels (ng/mg protein) in brain homogenates is depicted. These treatments exhibited a significant impact (F(3,20) = 17.46, *p* < 0.001) on COX-2 levels. Furthermore, the induction of LPS resulted in a notable increase (*p* < 0.001) in COX-2 levels (15.95 ± 1.006) in contrast to the control (9.261 ± 0.2849), indicating an upregulation of inflammatory reactions in the brain. Nevertheless, the administration of APZ led to a decrease (*p* < 0.001) in COX-2 levels in LPS-induced rats (1 mg/kg, 9.034 ± 0.7249; 2 mg/kg, 9.905 ± 0.9298), implying a reduction in the inflammatory response within the brain.

Regarding TNF-α levels (pg/mg protein) in the brain, both the APZ and LPS treatments induced significant modifications (F(3,20) = 3.950, *p* < 0.05) among the groups (Figure 3B). Following LPS treatment, rats exhibited significantly increased (*p* < 0.05) levels of TNF-α (419.0 ± 19.43) in comparison to the control group (343.6 ± 12.34). Regrettably, the elevated TNF-α levels induced by LPS did not show significant alterations following treatment with either of the two doses of APZ (1 mg/kg, 372.8 ± 12.78; 2 mg/kg, 376.7 ± 16.83).

The parameter NF-κB (ng/mg protein) was also significantly impacted (F(3,20) = 10.42, *p* < 0.001) by the treatments involving APZ and LPS. Specifically, the induction of LPS led to a substantial increase (14.05 ± 1.025, *p* < 0.01) in NF-κB levels within the brain when related to the control treatment (9.587 ± 0.3985). Interestingly, both the 1 mg/kg (10.77 ± 0.6924, *p* < 0.05) and 2 mg/kg (8.992 ± 0.5153, *p* < 0.001) doses of APZ led to a substantial decrease in NF-κB levels when matched to rats induced with LPS.

### 3.3. Evaluating the Influence of APZ and LPS on Oxidative Parameters

**Figure 4:**
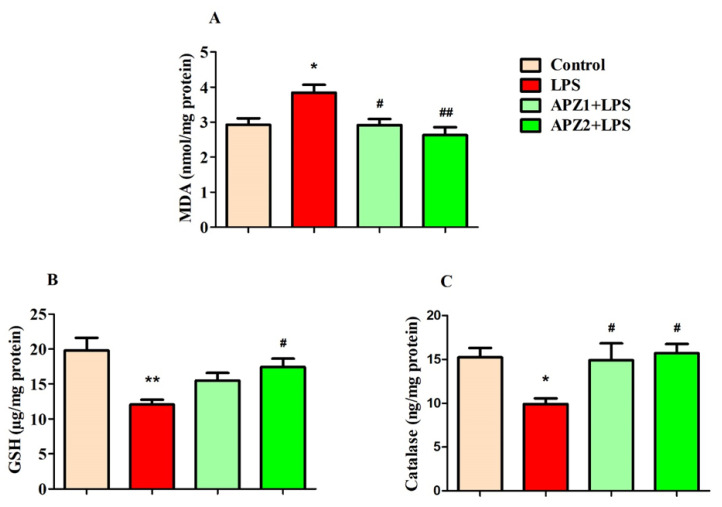
Effect of aripiprazole (APZ) on oxidative targets (**A**) MDA, (**B**) GSH, and (**C**) catalase in LPS-induced rats (mean ± SEM, *n* = 6). * *p* < 0.05, and ** *p* < 0.01 vs. control group; ^#^ *p* < 0.05, and ^##^ *p* < 0.01 vs. LPS-induced group.

Figure 4A–C depict the experimental results examining the impact of APZ and LPS on particular oxidative indicators, such as MDA, as well as the levels of antioxidants like GSH and catalase in the brain.

The statistical analysis identified a significant distinction between the treatment groups, as evident in the results (F(3,20) = 6.666, *p* < 0.01), particularly in relation to brain MDA (nmol/mg protein) levels (Figure 4A). Following the administration of four consecutive parenteral LPS injections, a noticeable increase in brain MDA levels (3.842 ± 0.2240; *p* < 0.05) was observed in relation to the control (2.927 ± 0.1870). Markedly, the administration of APZ treatments resulted in a dose-dependent decrease in LPS-induced MDA levels, with a statistically significant reduction seen at 1 mg/kg (2.918 ± 0.1723; *p* < 0.05) and a more pronounced reduction at 2 mg/kg (2.629 ± 0.2278; *p* < 0.01) within the brain.

When evaluating brain GSH levels (µg/mg protein), a statistically significant alteration (F(3,20) = 6.741; *p* < 0.01) was observed following both APZ and LPS treatments (Figure 4B). Specifically, the administration of LPS resulted in an increase of oxidative stress within brain tissues, characterized by a noteworthy decrease in GSH levels (12.05 ± 0.7012; *p* < 0.01) in contrast to the control (19.78 ± 1.813). Strikingly, the application of APZ, particularly at the higher dose of 2 mg/kg, resulted in a notable drop (*p* < 0.05) in GSH levels (17.43 ± 1.183) within the brains of rats subjected to LPS induction.

Simultaneously, levels of the second specific antioxidant, catalase (ng/mg protein), were notably impacted (F(3,20) = 6.741; *p* < 0.05) by both the administration of APZ and exposure to LPS (Figure 4C). It is important to mention that the rats exposed to LPS exhibited a moderate decrease (9.927 ± 0.6308; *p* < 0.05) in catalase levels parallel to the control group (15.26 ± 1.069). But when compared to the group only subjected to LPS, the thirty-day administration of APZ led to a substantial improvement (*p* < 0.05) in catalase levels (1 mg/kg; 14.92 ± 1.924 and 2 mg/kg; 15.72 ± 1.056) within the rat brain.

### 3.4. Evaluating the Influence of APZ and LPS on Apoptosis Parameters

**Figure 5:**
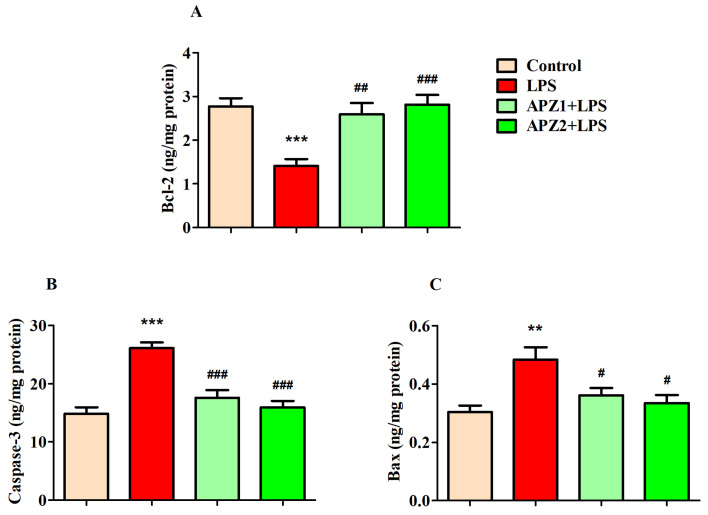
Effect of aripiprazole (APZ) on apoptosis proteins (**A**) Bcl-2, (**B**) caspase, and (**C**) Bax in LPS-induced rats (mean ± SEM, *n* = 6). ** *p* < 0.01, and *** *p* < 0.001 vs. control group; ^#^ *p* < 0.05, ^##^ *p* < 0.01, and ^###^ *p* < 0.001 vs. LPS-induced group.

Three critical parameters were examined to find the impact of both APZ and LPS on the rat brains: the anti-apoptosis marker Bcl-2 and the pro-apoptosis indicators Bax and Caspase-3 (Figure 5A–C).

The Bcl-2 levels (ng/mg protein) in the brain displayed substantial variations (F(3,20) = 10.39; *p* < 0.001) among the different treatment groups. Specifically, when comparing two particular groups, it was evident that the administration of LPS alone led to a considerable decrease (*p* < 0.001) in brain Bcl-2 levels (1.410 ± 0.1548) in relation to the control (2.772 ± 0.1867). Notably, these diminished Bcl-2 levels caused by LPS were effectively restored with the administration of APZ at both 1 mg/kg (2.596 ± 0.2511; *p* < 0.01) and 2 mg/kg (2.816 ± 0.2209; *p* < 0.001) in a dose-dependent manner (Figure 5A).

Notable alterations in the pro-apoptosis marker Caspase-3 levels (ng/mg protein) were evident (F(3,20) = 20.33; *p* < 0.001) following the APZ and LPS treatments in the rats’ brains. As anticipated, LPS induction led to a substantial increase in brain Caspase-3 levels (26.11 ± 0.9976; *p* < 0.001) when compared to the control (14.87 ± 1.102). By contrast, concurrent treatment with APZ (1 mg/kg; 17.60 ± 1.309 and 2 mg/kg; 15.97 ± 1.101) and LPS caused a reduction (*p* < 0.001) in brain Caspase-3 levels (Figure 5B).

In Figure 5C, a prominent influence (F(3,20) = 6.920; *p* < 0.01) of both APZ and LPS on brain Bax levels (ng/mg protein) is depicted. It is important to highlight the substantial rise (*p* < 0.01) in Bax levels (0.4837 ± 0.0419) in the brains of the rats induced with LPS, as compared to the control rats (0.3033 ± 0.0226). Interestingly, a reduction (*p* < 0.01) in Bax levels was observed following the treatment with APZ at 1 mg/kg (0.3615 ± 0.0245) and 2 mg/kg (0.3345 ± 0.0270) in comparison to the LPS treatment (Figure 5C).

## 4. Discussion

Recent research has shown a growing interest in atypical antipsychotic drugs for their potential to enhance cognitive functions in individuals undergoing treatment for schizophrenia. One such novel atypical antipsychotic, referred to as APZ, has demonstrated promising effects on cognitive improvement in individuals diagnosed with schizophrenia or schizoaffective disorder [9,10,11]. Our study explored the impact of APZ on spatial recognition memory, and the findings of this study unveiled potential neuroprotective mechanisms associated with APZ, particularly in countering neuroinflammation, oxidative stress, and apoptosis triggered by LPS-induced neurotoxicity. Significantly, the results of this investigation provided support for the enhanced recognition memory of rats in the NOR test and the mitigation of neurotoxic mechanisms induced by LPS. This underscores the potential of APZ as a valuable asset in ameliorating cognitive deficits and protecting against neurotoxicity associated with conditions like schizophrenia.

Cognitive deficits and memory impairments are commonly regarded as fundamental aspects of schizophrenia. Impaired neurogenesis of brain neurons is thought to be a significant contributor to cognitive deficits in neuropsychiatric illnesses including schizophrenia and depression [26]. Fortunately, there is evidence suggesting the potential improvement of various cognitive functions in individuals with psychosis through APZ treatment. Specifically, APZ therapy in schizophrenic patients has been shown to significantly improve verbal cognitive functioning [9]. Additionally, APZ has demonstrated the capacity to enhance social cognition and neurocognition in individuals diagnosed with schizophrenia [10]. Furthermore, a long-acting formulation of APZ has demonstrated the ability to enhance cerebral blood flow in the right frontal and temporal regions, along with improvements in cognitive scores among patients with schizophrenia [11].

Our current research has incorporated NOR tasks to assess the cognitive capabilities of rats administered with APZ, with a specific focus on their object recognition memory concerning two distinct objects in the context of memory deficits induced by LPS exposure. The LPS model is recognized for its association with neurodegeneration akin to the pathology seen in Alzheimer’s and Parkinson’s diseases. The current findings validate that inducing LPS in rats leads to significant impairment in the NOR test. Specifically, LPS administration resulted in a marked decrease in animals’ exploration of both the FO and the NO, as well as their ability to discriminate between these objects, quantified by the DI%. However, the treatment with APZ resulted in enhanced exploration times for both the FO and the NO. Notably, it improved the discrimination ability, as reflected in higher DI% values during the test sessions. Interestingly, the recent animal experiments have provided additional evidence supporting the positive influence of APZ on the enhancement of cognitive functions. The co-administration of APZ and a nerve growth factor resulted in enhanced cognitive performance in open-field and Morris water maze (MWM) tests within a mouse model of schizophrenia induced by dizocilpine [12]. The concurrent administration of APZ and cilostazol provided additional evidence for the enhancement of memory and cognitive performance in MWM among mice with vascular dementia [14]. Moreover, in animals with induced schizophrenia who had also been exposed to tobacco smoke, there was an improved spatial memory performance in the MWM following APZ treatment [15].

In the context of understanding the pathophysiology of psychosis, neuroinflammation and oxidative stress play pivotal roles, and their intricate interconnections are evident. In particular, the inflammatory processes within neuronal cells have been closely associated with various neurodegenerative pathways, which in turn are linked to the onset of neurodegenerative conditions, psychosis, and depression. In schizophrenia, neuroinflammation denotes the initiation of the brain’s immune response, which is principally driven by microglial cells and the discharge of inflammatory cytokines like IL-2, IL-4, IL-13 IFN-γ, and TNF-α. This elevated inflammatory state can be triggered by diverse factors, including infection, stress, or autoimmune conditions [16,17,27,28]. Moreover, neuroinflammation has the capacity to disturb neural circuits and impact neurotransmitter systems, which could potentially lead to the manifestation of psychotic symptoms [28].

In the current study, a widely recognized inflammatory-inducing agent, LPS, is employed to induce neurotoxicity. Numerous investigations have indicated that exposure to LPS triggers the release of various vulnerable cytokines such as IL-1α, IL-6, TNF-α, and IL-1β [29]. In a similar vein, our research observed a rise in TNF-α, COX-2, and NF-κB levels in the brains induced by LPS. Unexpectedly, the administration of APZ did not mitigate the rise in TNF-α levels induced by LPS in the brain. In a positive outcome, the administration of APZ led to a noteworthy reduction in COX-2 and NF-κB levels in brain tissues, indicating effective inhibition of their production. Further, recent studies have documented the anti-inflammatory efficacy of APZ. In peptidoglycan-stimulated RAW264.7 macrophages, APZ effectively reduced the mRNA expression levels of inflammatory genes, such as COX-2, iNOS, and TNF-α. Furthermore, APZ exhibited the ability to suppress various molecular pathway mediators associated with inflammation, including NF-κB [30]. In addition, APZ treatment resulted in the decrease of proinflammatory cytokine production, encompassing TNF-α, IL-1β, IL-2, IL-6, IL-7, IL-13, IL-18, IL-21, and IL-23, in both psychiatric patients and human immune cells [16,18,31].

Oxidative stress leads to inflammatory mediators and a series of pathways that trigger inflammation, ultimately resulting in memory impairment [32]. In schizophrenia, elevated levels of stress biomarkers have been detected in peripheral blood, as well as in its components, cerebrospinal fluids, and brain tissues [33]. There is an imbalance in the oxidative system, with the endogenous antioxidant GSH playing a crucial role in counteracting oxidative stress and protecting tissues from excessive damage. Depletion of GSH levels in the brain is correlated with the development of psychosis symptoms and is additionally linked to deficiencies in NMDA receptor functions [34]. Moreover, an up-to-date collective report emphasizes the association between schizophrenia and oxidative stress markers, which includes a decline in antioxidant levels such as GSH and catalase, coupled with an elevation in markers of lipid peroxidation (LPO) in the peripheral system [35].

In our study, we found that rats injected with LPS displayed a rise in oxidative burden, as identified by higher levels of MDA, a marker of LPO, and a decline in antioxidant capacity, which was characterized by decreased levels of GSH and catalase in the brain. Interestingly, APZ was found to alleviate the oxidative vulnerability induced by LPS by lowering MDA levels and increasing GSH and catalase levels in the brain. In matching mice induced with manic symptoms, APZ demonstrated its antioxidant properties by influencing the levels of several oxidative-related biomarkers in the brain, including LPO, catalase, GSH, superoxide dismutase, and glutathione peroxidase [22]. Additionally, APZ successfully mitigated oxidative stress in rats with autism by normalizing the GSH and catalase levels while simultaneously reducing MDA in the targeted brain regions investigated [21].

In neurodegenerative conditions, oxidative stress has been linked to apoptosis-related cell death. Within neurons, increased production of ROS disrupts mitochondrial functions, initiating a destructive cycle of ROS-induced damage, and ultimately triggering apoptosis in neuronal cells [36]. Considering the intrinsic apoptosis pathway, Bax leads its pro-apoptotic functions by releasing Cyto c from cellular mitochondria when stimulated by external pro-apoptotic signals. Released Cyto c then causes the cleavage of Procaspase-3 to its active form, Caspase-3. This active form results in cellular death, involving the breakdown of cellular components and DNA damage. The anti-apoptotic protein Bcl-2 counteracts this mechanism by preventing Bax-mediated Cyto c release from the mitochondria. In contrast, TNF-α binds to death receptors (extrinsic apoptosis pathway) on the cell membrane, initiating the activation of Caspase-8, which subsequently activates Caspase-3 from Procaspase-3, leading to cell death [36].

In our present results, following the successful administration of four peripheral LPS injections, a decrease in Bcl-2 levels and a simultaneous rise in both Caspase-3 and Bax levels in the brain were noted, indicating an increased incidence of neuronal apoptosis. However, oral administration of APZ exhibited the capacity to alleviate LPS-resulted apoptosis by enhancing its anti-apoptotic function via elevating Bcl-2 levels. Furthermore, it countered the apoptotic process by regulating pro-apoptotic activities, resulting in changes in Caspase-3 and Bax levels in the brains of LPS-induced rats.

In schizophrenia, elevated apoptosis in specific brain regions may lead to neuronal death, contributing to cognitive and functional impairments in individuals with the condition. An investigation of brain tissue from schizophrenia patients identified a decrease in Bcl-2 levels (around 25% when compared to normal) in the middle temporal gyrus (MTG) [37]. Additionally, the latest study has emphasized the role of Caspase activity in the onset of schizophrenia. This study unveiled elevated gene expression levels of both Caspase-3 and Caspase-9 in peripheral blood samples obtained from patients, further underscoring the importance of Caspases in this particular context [38]. On a positive note, APZ exhibited its neuroprotective capabilities by means of its anti-apoptotic function. It serves to protect neurons by preventing excessive glutamate-induced apoptotic cell death in rats [39]. Furthermore, APZ exhibited its neuroprotective properties in SH-SY5Y human neuroblastoma cells by increasing both BDNF and Bcl-2 levels [40]. These findings provide additional support for our study, indicating that APZ has a defensive effect against LPS-induced neuronal apoptosis.

## 5. Conclusions

Our cumulative results imply that APZ mitigates LPS-induced memory deficits and neuronal toxicity in rats by protecting against neuroinflammation, diminishing oxidative stress, and suppressing cellular apoptosis mechanisms. APZ treatment improved spatial recognition memory in the NOR test by enhancing the performance in object exploration. Our understanding is that the neuroprotective effect elicited by APZ may be linked to its ability to decrease markers associated with inflammation (COX-2 and NF-κB), oxidative stress (MDA), and cellular apoptosis (Caspase-3 and Bax). Moreover, APZ elevated the levels of antioxidants (GSH and catalase) and enhanced anti-apoptotic activity (Bcl-2) in the rat brains. These promising outcomes lend support to the potential therapeutic application of APZ for addressing cognitive decline in conditions related to psychosis and neurodegenerative disorders. Nevertheless, further extensive experimentation is needed to uncover the specific mechanism responsible for the neuroprotective effects of APZ.

## Data Availability

The data presented in this study are available from the corresponding author upon reasonable request.
